# Taking advantage of an unerupted third molar: a case report

**DOI:** 10.1590/2177-6709.22.4.097-101.oar

**Published:** 2017

**Authors:** Igor Figueiredo Pereira, Fernando Zander Mucci Santiago, Augusto Cesar Sette-Dias, Vladimir Reimar Augusto de Souza Noronha

**Affiliations:** 1 Universidade de Pernambuco, Faculdade de Odontologia (Recife/PE, Brasil).; 2 Universidade Federal de Minas Gerais, Faculdade de Odontologia (Belo Horizonte/MG, Brasil).; 3 Universidade Federal de Minas Gerais, Faculdade de Odontologia, Centro de Ciências da Saúde, Departamento de Clínica, Patologia e Cirurgia Odontológicas (Belo Horizonte/MG, Brasil).

**Keywords:** Impacted tooth, Unerupted tooth, Third molar.

## Abstract

**Introduction:**

Treatments with dental surgery seek to displace tooth to the correct position within the dental arch.

**Objective:**

To report a clinical case that took advantage of an unerupted third molar.

**Case history:**

A male patient, 18 years of age, was referred by his dentist to evaluate the third molars. The clinical exam revealed no visible lower third molars. The computed tomography (CT) exam showed the presence of a supernumerary tooth in the region of the mandibular ramus, on the left side, and impaction of the third molar, which was causing root resorption on the second molar, thus making it impossible to remain in the buccal cavity. The preferred option, therefore, was to remove both second molar and the supernumerary tooth, in addition to attaching a device to the third molar during surgery for further traction.

**Results:**

After 12 months, the third molar reached the proper position.

**Conclusion:**

When a mandibular second permanent molar shows an atypical root resorption, an impacted third molar can effectively substitute the tooth by using an appropriate orthodontic-surgical approach.

## RESUMO

**Introdução:**

o objetivo do tratamento odontológico cirúrgico é deslocar os dentes para a posição correta na arcada dentária.

**Objetivo:**

relatar um caso clínico de aproveitamento de terceiro molar não irrompido.

**Histórico do caso:**

paciente do sexo masculino, com 18 anos de idade, foi encaminhado, pelo dentista responsável pelo caso, para avaliação dos terceiros molares. Ao exame clínico, os terceiros molares inferiores não eram visíveis. Com a tomografia computadorizada (TC), foi possível identificar a presença de um dente supranumerário na região do lado esquerdo do ramo da mandíbula, além de impacção do terceiro molar, causadora de reabsorção radicular no segundo molar, tornando impossível a permanência desse dente na cavidade bucal. Logo, optou-se pela remoção tanto do segundo molar quanto do dente supranumerário, além da instalação de um aparelho no terceiro molar, durante o procedimento cirúrgico, para posterior tracionamento.

**Resultados:**

após 12 meses, o terceiro molar alcançou a posição adequada.

**Conclusão:**

nos casos em que o segundo molar inferior permanente apresenta reabsorção radicular atípica, um terceiro molar impactado pode substituir o dente reabsorvido, de maneira eficaz, através de uma abordagem ortocirúrgica apropriada.

## INTRODUCTION

Dental eruption occurs with great precision in the great majority of human beings. The deciduous and permanent teeth are formed inside the maxillary bone and, at a certain point, erupt to fully develop chewing and speech functions.^1^

Failure on dental eruption is a common clinical situation that represents an unfavorable aspect from the esthetic and functional points of view. Among the regions that are affected by this problem, a greater occurrence is observed in the third molars and canines, but this event can also be found in other regions of the oral cavity.^2^

The third molars are most frequently impacted (20 to 30%) because they are the last teeth to erupt in the oral cavity, followed by the maxillary canines (most with palatal dislocation), mandibular second premolars, and central maxillary incisors.^3^

Orthodontic traction of an impacted tooth can be performed in many ways, including: the direct attachment of brackets, buttons, or wires to the retained teeth and with an application of force placed on fixed and removable appliances. The anchorage of fixed devices offers a greater control and effectiveness of the applied force, thus minimizing undesired effects, although they may still occur.^1^

Few studies in the literature report on the orthodontic traction of third molars. Nevertheless, in some cases, taking advantage of third molars shows a good alternative to compensate second molar loss. Orthodontists should be aware of the third molars, even when these teeth do not interfere directly in the planning and treatment.^4^

Therefore, the present study sought to report a case that took advantage of an unerupted mandibular third molar.

## CASE HISTORY

A male patient, 18 years of age, sought the Surgery and Oral/Maxillofacial Traumatology service for an evaluation of third molars. The patient was referred by the orthodontist, who requested the extraction of lower left second molar, due to root resorption caused by impaction of the third molar crown. The extraction of the supernumerary tooth located in the region of the mandibular ramus on the left side was also indicated. Then, the patient underwent an orthodontic traction of the third molar.

In the overall evaluation, the patient reported no systemic changes or the use of any type of medications.

**Figure 1:**
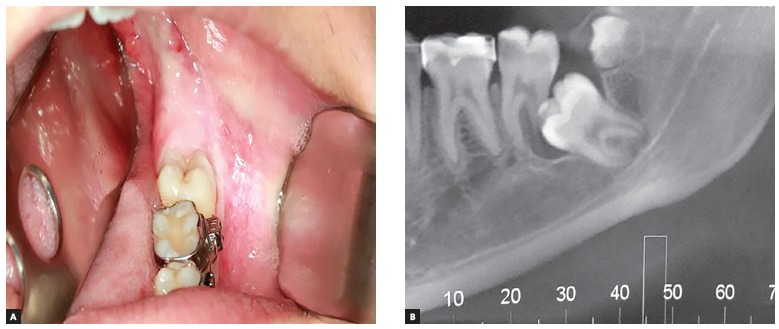
Pre-operative aspect, left posterior mandibular region: A) Clinical aspect; B) CT image, showing unerupted tooth #38 and the supernumerary tooth, as well as the root resorption of tooth #37.

After undergoing an extraoral exam, the patient presented no visible or palpable changes. In the intraoral evaluation, the absence of tooth #38 was observed. A subsequent computed tomography (CT) exam showed that the tooth #38 was unerupted and impacted by tooth #37. The accentuated resorption of the distal root indicated the tooth for extraction (Fig 1).

**Figure 2:**
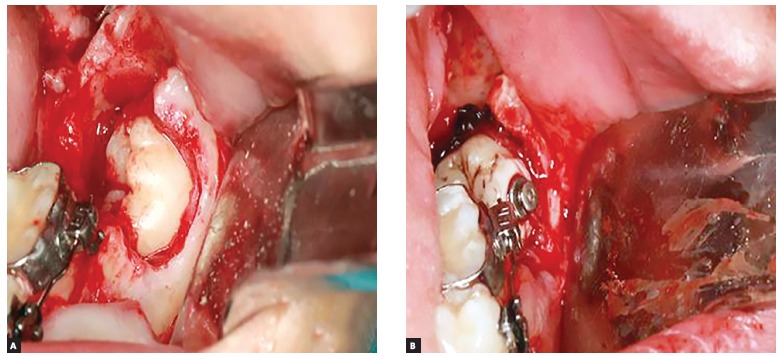
Trans-operative aspect: A) image after the removal of tooth #37 and the supernumerary tooth, as well as the exposure of tooth #38; B) attachment of orthodontic device on tooth #38.

In this case, an orthodontic surgical treatment was recommended, opting for the removal of tooth #37 and the supernumerary tooth. In addition, a button was bonded onto the buccal surface of the tooth #38, which was anchored through a wire (0.3mm) to the orthodontic appliance, for further orthodontic traction of the tooth #38 (Fig 2).

The procedure was performed under local anesthesia, using 2% lidocaine with vasoconstrictor. A conventional incision was made to remove the third molar, with total mucoperiosteal displacement, for the appropriate exposure of the teeth. After the procedure, the patient was medicated with antibiotics for seven days, and anti-inflammatory and analgesic medications for three days.

**Figure 3:**
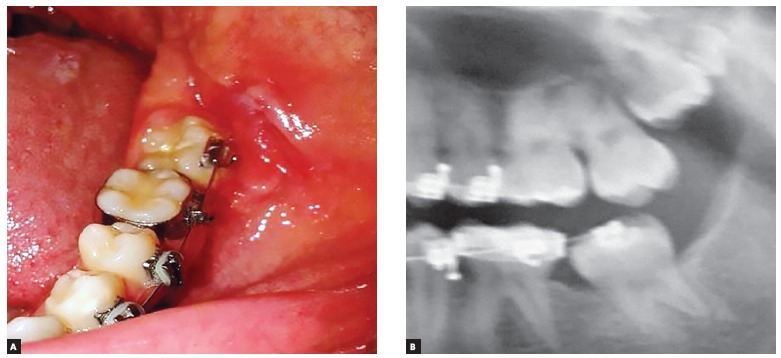
Aspect after 12 months, showing the eruption of tooth #38: A) clinical aspect; B) radiographic image.

A detailed follow-up of the patient was performed. After 12 months, tooth #38 was observed in its proper position within the dental arch (Fig 3).

## DISCUSSION 

Patients must be aware of all risks inherent to their treatment and should be well-advised by the dentist. All possibilities should be discussed in advance, including possible complications, especially in orthodontic-surgery combined treatments.

Tooth impactions occur as a result of displacements in the normal sequence of occlusion development. In addition, impacted teeth can cause various problems, such as roots resorption of neighboring teeth, a loss of length in the dental arch, the formation of a dentigerous cyst, local infections, reflex pain, etc. In the present case, tooth #38 caused the loss of tooth #37 due to the resorption of the distal root. The tooth submitted to traction presented no divergent roots, root lacerations or other conditions that contraindicated orthodontic traction. The technical difficulty in achieving the proper force to tractioning teeth with divergent roots contraindicates this procedure.^5^

Another point that should be observed is the periodontal tissues condition, since the tooth will be displaced great distances for a long period of time. For this reason, it is essential to maintain a good oral hygiene, in such a way that the alveolar bone and the soft tissue follow along the tooth, in order to prevent gingival recession. The present patient was quite cooperative and aware of his role in the treatment results, presenting a favorable oral hygiene, with no history of periodontal problems.^6^ ^,^ ^7^

The appropriate removal of the bone tissue surrounding the crown of the pulled tooth constitute an important step toward a successful treatment. In the present case, an osteotomy was performed in the cementoenamel junction, for the correct insertion of the orthodontic device, providing a better distribution of the orthodontic forces.^8^

Well-planned and executed orthodontic traction represents a safe procedure whose minimal consequences are clinically controllable. Thus, this is a safe procedure, with minimal consequences. Even when associated with surgically induced dislocation, which is also well-planned and conscientiously executed, orthodontic traction still represents a safe procedure.^8^

It is not common taking advantage of unerupted and impacted third molar, due to location and difficult traction. Few reports show the treatment with successful results, especially due to the inappropriate exposure of the crown and the improper application of orthodontic traction. This type of problem leads many professionals to fail to take full advantage of this procedure.^9^

It is also important to remember that taking advantage of this tooth allows correct homeostasis within the region, regardless of prosthetic rehabilitation, which would certainly promote a lasting and effective rehabilitation, without the need for future interventions.^8^ This fact was observed in the present case, of a young patient who would lose a significant portion of bone in the region surrounding the tooth, which would hinder a later rehabilitation with implants.

In the present case, CT exam enabled to verify, with greater precision, the relationship between the impacted third molar and the adjacent teeth and noble structures.^3^

The CT exam, in this case, was very important as a diagnostic aid. This exam provided a better observation of the relationship of tooth #38 with the noble structures, such as the inferior alveolar nerve, as well as its relationship with the distal surface of tooth #37. The morphology of the root and crown of tooth #38 were also observed, thus determining the viability of the orthodontic traction of this tooth. In this context, the chances of complications in the trans-operative period and during orthodontic traction were reduced.^10^

Previous studies reported means of performing orthodontic traction of unerupted teeth, such as the attachment of orthodontic devices, buttons or brackets, and the use of fixing plates for anchoring.^11^ In the present clinical case, an orthodontic button was used, which is described as a simple technique that causes less morbidity for the patient. Maintaining the teeth in the arch is important, to ensure that the patient will have adequate functionality and good aesthetics.^11^ ^,^ ^12^

## FINAL CONSIDERATIONS

The use of tooth #38 was considered to be quick and effective. However, what should be taken into consideration is the importance of having sufficient space in the dental arch and a favorable position of the impacted tooth before beginning such treatment. The prognosis will depend on the patient’s age, in addition to complementary exams, to determine the position and the possible complication that can hinder orthodontic traction. Hence, when a mandibular second permanent molar shows an atypical root resorption, an impacted third molar can effectively substitute the tooth by using an appropriate orthodontic-surgical approach.
